# No evidence for HPAI H5N1 2.3.4.4b incursion into Australia in 2022

**DOI:** 10.1111/irv.13118

**Published:** 2023-03-08

**Authors:** Michelle Wille, Marcel Klaassen

**Affiliations:** ^1^ Sydney Institute for Infectious Diseases, School of Medical Sciences The University of Sydney Sydney New South Wales Australia; ^2^ Department of Microbiology and Immunology, at the Peter Doherty Institute for Infection and Immunity The University of Melbourne Melbourne Victoria Australia; ^3^ WHO Collaborating Centre for Reference and Research on Influenza, at the Peter Doherty Institute for Infection and Immunity Melbourne Victoria Australia; ^4^ Centre for Integrative Ecology Deakin University Geelong Victoria Australia; ^5^ Victorian Wader Study Group Thornbury Victoria Australia; ^6^ Australasian Wader Studies Group Curtin ACT Australia

**Keywords:** Australia, avian influenza, H5N1, HPAI, influenza A virus

## AUTHOR CONTRIBUTIONS

**Michelle Wille:** Conceptualization; formal analysis; investigation; methodology; project administration; writing ‐ original draft; writing ‐ review and editing. **Marcel Klaassen:** Conceptualization; formal analysis; investigation; methodology; writing ‐ original draft; writing ‐ review and editing.

## CONFLICT OF INTEREST

The authors declare no conflict of interest.

### PEER REVIEW

The peer review history for this article is available at https://publons.com/publon/10.1111/irv.13118.

The current high pathogenicity avian influenza (HPAI) H5 panzootic is having a profound impact on the poultry industry and wildlife.^1^ While lineage 2.3.4.4b is of current concern, HPAI H5 emerged in poultry in 1996 and has caused outbreaks in wild bird populations episodically since 2005.^2^ The epidemiology of this virus has changed substantially with the emergence of new lineages, as exampled by Clade 2 viruses that caused the first wild bird mass mortality event at Qinghai Lake, China in 2005.^3^ A novel lineage emerged in 2014 (2.3.4.4), which has diversified and caused substantial mortality, including mass mortality events of wild birds in 2014, 2016 and 2021–present, along with ongoing outbreaks in poultry in Eurasia and North America.^2^

Understanding viral incursion risk following the emergence of novel lineages of HPAI with their own specific phenotype is of crucial importance in preventing incursion events, improving biosecurity to protect poultry and responding to wild bird outbreaks. The viral incursion into North America in December 2021 was not detected until outbreaks occurred in poultry.^4^ The recent incursion into South America, in November 2022, was only detected following mass mortality events.^5^ Wild migratory waterfowl have been predominantly implicated in the re‐occuring incursions into Europe and Africa.^6^ However, there are few migratory waterfowl linking the Nearctic and Palearctic, as well as North and South America, suggesting that the long‐distance dispersal of lineage 2.3.4.4b HPAI may rely on additional bird groups other than waterfowl (e.g., Günther et al.^7^).

**FIGURE 1:**
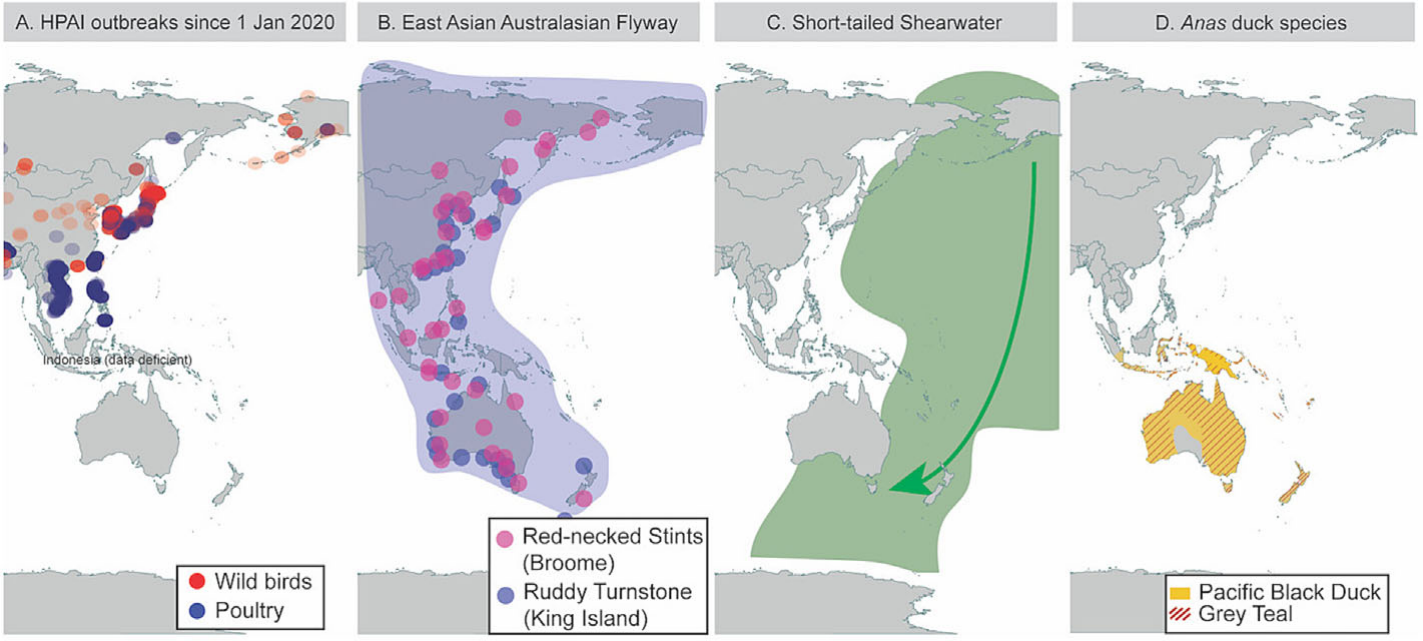
HPAI outbreaks along the East Asian Australasian flyway and distributions of key avian influenza reservoir species in Australia. (A) Outbreaks of HPAI in wild birds (red symbols where intensity reflects number of outbreaks at that location) and poultry (blue symbols) since 1 January 2020. Data mined from the World Animal Health Information System of the World Organisation for Animal Health at https://wahis.woah.org/. (B) The East Asian Australasian Flyway utilised by migratory shorebirds. Migratory propensity is exemplified for two populations that we sampled most intensively: Red‐necked Stints originally colour‐marked in Broome, Western Australia (purple symbols), and Ruddy Turnstones originally marked on King Island, Tasmania (blue symbols). Data extracted from https://www.birdmark.net/. (C) Distribution of Short‐tailed Shearwater. Arrow demonstrates southbound migration to Australia occurs from Beringia. (D) Map illustrating the contrasting and limited, Australo‐papuan distribution of Australian waterfowl using the distribution of Pacific Black Duck ( *Anas superciliosa* ) and Grey Teal ( *Anas gracilis* ) as an example. All duck species found in Australia are endemic to Australio‐Papuan region and do not migrate to Asia; hence, they are likely to play a nominal role in viral incursions from Asia and were therefore not prioritised in this study.

Lineage 2.3.4.4b has now been detected on all continents except Australia and Antarctica.^8^ HPAI incursion risk to Australia has previously been considered low due to the absence of waterfowl species that migrate beyond Australia^9^ (Figure 1), as also exemplified from influenza genomic surveillance.^10^ Still, annually, millions of migratory seabirds and shorebirds migrate from Asia and North America to Australia (Figure 1). Some of these species have been shown to be part of the avian influenza reservoir community^11^ and potentially survive and move HPAI viruses.^12^

To reveal whether a viral incursion may have occurred in Australia in 2022 with the arrival of wild migratory sea‐ and shorebirds, we investigated 817 migratory birds of the order *Charadriiformes* and *Procelariformes*, in September–December 2022. Specifically, we captured and sampled Short‐tailed Shearwaters (*Puffinus tenuirostris*, *n* = 233) upon their arrival from the northern Pacific to a breeding colony on Philip Island, Victoria, and 12 Asian‐breeding migratory shorebird species at major non‐breeding sites in Roebuck Bay and 80 mile beach, Western Australia (*n* = 509) including Bar‐tailed Godwit (*Limosa lapponica*, *n* = 72), Black‐tailed Godwit (*Limosa limosa*, *n* = 14), Curlew Sandpiper (*Calidris ferruginea*, *n* = 23), Great Knot (*Calidris tenuirostris*, *n* = 71), Red Knot (*Calidris canutus*, *n* = 45), Red‐necked Stint (*Calidris ruficollis*, *n* = 102), Sanderling (*Calidris alba*, *n* = 3), Ruddy Turnstone (*Arenaria interpres*, *n* = 25), Grey‐tailed Tattler (*Tringa brevipes*, *n* = 50), Terek Sandpiper (*Xenus cinereus*, *n* = 50), Greater Sandplover (*Charadrius leschenaultia*, *n* = 49), Lesser Sandplover (*Charadrius mongolus*, *n* = 3) and Gull‐billed Tern (*Gelochelidon nilotica*, *n* = 1). Finally, we also sampled Ruddy Turnstones at a non‐breeding site on King Island, Tasmania (*n* = 75) (Figure 1). Capture, banding and sampling were conducted under Victorian Wader Study Group's ABBBS authority 8001, Deakin University animal ethics committee (B39‐2019), Department of Primary Industries and Regional Development WA (20‐4‐10) and Department of Natural Resources and Environment (5/2019–2020).

All samples were negative for influenza A virus by qPCR, following Wille et al.^11^ Twenty‐five serum samples tested positive for anti‐NP antibodies using a commercial ELISA (given an S/N cut off of 0.5), which fell within the previously reported seroprevalence of the species that tested positive^11^: Red‐necked Stint (8/102), Red Knot (4/45), Ruddy Turnstone (3/75) and Short‐tailed Shearwater (10/231). All sera samples positive by anti‐NP ELISA were negative on a subsequent hemagglutination inhibition (HI) assay using a lineage 2.3.4.4b candidate vaccine virus A/Astrakhan/3212/2020(H5N8)^13^ following Wille et al.^12^ A candidate vaccine virus is a 6:2 recombinant virus on an A/Puerto Rico/8/1934(H1N1)(PR8) backbone with the multi‐basic cleavage site removed. In addition to the absence of HPAI and antibodies against HPAI lineage 2.3.4.4.b in the sampled migrants, there were neither indications of increased mortality in any wild birds nor reports of unusual mortality in poultry across Australia.

For Australia as for other regions in the world, HPAI incursion risk hinges on a combination of factors, including wild bird migration, virus pathogenicity in wild birds (notably whether wild birds are able to migrate while infected) and outbreaks and virus circulation in neighbouring regions (particularly at key stopover sites for migratory birds). That there was no incursion of HPAI in Australia in 2022 despite the arrival of millions of migratory birds, the capacity of wild birds to disperse this virus large distances (e.g., Caliendo et al.^4^), the apparent widening of the virus' host reservoir beyond waterfowl^7^, ^8^, ^14^ and high levels of HPAI activity in Asian countries along the East Asian Australasian flyway^8^ is unclear and warrants further investigation.

As the spatial distribution and intensity of HPAI H5 outbreaks in birds has increased, we have seen a corresponding increase in the number of mammalian cases, including human cases.^1^ There has also been indication of mammal‐to‐mammal transmission for the first time since the emergence of this lineage,^15^ such that this avian panzootic has important implications for humans. Australia will again enter a high‐risk period when the major bird migrations into the country take place between August and November 2023. Continued surveillance is critical for early detection and rapid response, and as such, we call for enhanced surveillance of Australian wild birds to match heightened incursion risk in the second half of 2023.

## Data Availability

The data that support the findings of this study are available from the corresponding author upon reasonable request.
